# Resveratrol: A Double-Edged Sword in Health Benefits

**DOI:** 10.3390/biomedicines6030091

**Published:** 2018-09-09

**Authors:** Bahare Salehi, Abhay Prakash Mishra, Manisha Nigam, Bilge Sener, Mehtap Kilic, Mehdi Sharifi-Rad, Patrick Valere Tsouh Fokou, Natália Martins, Javad Sharifi-Rad

**Affiliations:** 1 Medical Ethics and Law Research Center, Shahid Beheshti University of Medical Sciences, Tehran 88777539, Iran; bahar.salehi007@gmail.com; 2 Student Research Committee, Shahid Beheshti University of Medical Sciences, Tehran 22439789, Iran; 3 Department of Pharmaceutical Chemistry, H. N. B. Garhwal (A Central) University, Srinagar Garhwal 246174, Uttarakhand, India; abhaypharmachemhnbgu@gmail.com; 4 Department of Biochemistry, H. N. B. Garhwal (A Central) University, Srinagar Garhwal 246174, Uttarakhand, India; m.nigam@hnbgu.ac.in; 5 Gazi University Faculty of Pharmacy Department of Pharmacognosy, Ankara 06330, Turkey; bilgesener11@gmail.com (B.S.); klcmehtap89@gmail.com (M.K.); 6 Department of Medical Parasitology, Zabol University of Medical Sciences, Zabol 61663335, Iran; 7 Antimicrobial and Biocontrol Agents Unit, Department of Biochemistry, Faculty of Science, University of Yaounde 1, Ngoa Ekelle, Annex Fac. Sci, P.O. Box. 812, Yaounde-Cameroon; 8 Faculty of Medicine, University of Porto, Alameda Prof. Hernâni Monteiro, Porto 4200-319, Portugal; 9 Institute for Research and Innovation in Health (i3S), University of Porto, Porto 4200-135, Portugal; 10 Phytochemistry Research Center, Shahid Beheshti University of Medical Sciences, Tehran 11369, Iran; 11 Department of Chemistry, Richardson College for the Environmental Science Complex, The University of Winnipeg, Winnipeg, MB R3B 2G3, Canada

**Keywords:** resveratrol, physiological effects, pharmacological activity, antioxidant, anticancer, antimicrobial

## Abstract

Resveratrol (3,5,4′-trihydroxy-trans-stilbene) belongs to polyphenols’ stilbenoids group, possessing two phenol rings linked to each other by an ethylene bridge. This natural polyphenol has been detected in more than 70 plant species, especially in grapes’ skin and seeds, and was found in discrete amounts in red wines and various human foods. It is a phytoalexin that acts against pathogens, including bacteria and fungi. As a natural food ingredient, numerous studies have demonstrated that resveratrol possesses a very high antioxidant potential. Resveratrol also exhibit antitumor activity, and is considered a potential candidate for prevention and treatment of several types of cancer. Indeed, resveratrol anticancer properties have been confirmed by many in vitro and in vivo studies, which shows that resveratrol is able to inhibit all carcinogenesis stages (e.g., initiation, promotion and progression). Even more, other bioactive effects, namely as anti-inflammatory, anticarcinogenic, cardioprotective, vasorelaxant, phytoestrogenic and neuroprotective have also been reported. Nonetheless, resveratrol application is still being a major challenge for pharmaceutical industry, due to its poor solubility and bioavailability, as well as adverse effects. In this sense, this review summarized current data on resveratrol pharmacological effects.

## 1. Introduction

Among many phytochemicals, phytoestrogens have been reported to contain several bioactive molecules, mostly found in soy, vegetables and fruits. These compounds can be classified into four main groups, such as isoflavonoids, flavonoids, stilbenes and lignans. From them, stilbenes, in particular *trans*-resveratrol and its glucoside, are widely reported to be beneficial to human health, having even shown to possess antioxidant, anticarcinogenic, antitumor and estrogenic/antiestrogenic activity [1].

Specifically, resveratrol is well known biologically active compound synthesized by plants undergoing infectious or ionizing radiation. Renaud and De Lorgeril were the first to relate wine polyphenols such as resveratrol, to the potential health benefits attributed to regular and moderate wine consumption (the so called “French Paradox”) [2]. Resveratrol has since received an increasing scientific attention, leading to investigation on its biological activity, and to numerous publications [3]. Resveratrol was first isolated from white hellebore (*Veratrum grandiflorum* O. Loes) roots in 1940, then from *Polygonum cuspidatum* roots in 1963, a plant used in traditional Chinese and Japanese Medicine as anti-inflammatory and anti-platelet agent. This natural polyphenol has been detected in more than 70 plant species, and is also found in discrete amounts in red wines and various human foods. High concentrations are present in grapes, possibly because of *Vitis vinifera* response to fungal infection. In plants, resveratrol acts as a phytoalexin that is synthesized in response to mechanical injury, UV irradiation and fungal attacks. For industrial purposes, resveratrol is generally obtained by chemical or biotechnological synthesis from yeasts *Saccharomyces cerevisiae* [4,5,6,7,8].

As of today, 92 new resveratrol compounds, including 39 dimers, 23 trimers, 13 tetramers, 6 resveratrol monomers, 6 hexamers, 4 pentamers, and 1 octamer have been reported from the *Dipterocarpaceae*, *Paeoniaceae*, *Vitaceae*, *Leguminosae*, *Gnetaceae*, *Cyperaceae*, *Polygonaceae Gramineae*, and *Poaceae* families [9]. Among these families, *Dipterocarpaceae*, containing 50 resveratrol’s, accounts for the majority, being involved 7 *Dipterocarpaceae* genera, including *Vatica*, *Vateria*, *Shorea*, *Hopea*, *Neobalanocarpus*, *Dipterocarpus* and *Dryobalanops* [9]. Currently, resveratrol is sold as a nutritional supplement with a wide range of pharmacological effects, including cellular defensive action against oxidative stress [10,11,12]. In this sense, the present review summarizes resveratrol’ beneficial health effects, including anticancer, antimicrobial, neuroprotective, antiaging, anti-inflammatory, cardioprotective and blood-sugar lowering properties, as also life-prolonging effects.

## 2. Chemistry of Resveratrol

**Figure 1:**
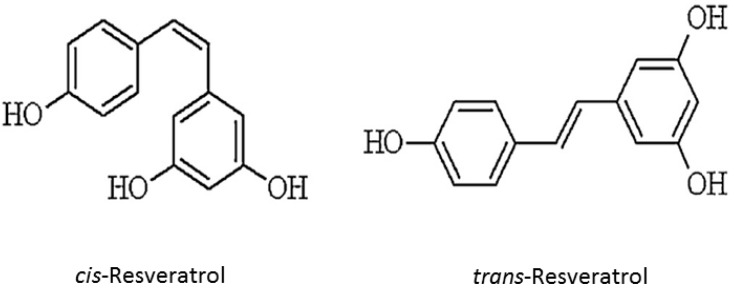
Resveratrol chemical structure (*cis* and *trans* forms).

Resveratrol is a stilbenoid polyphenol, possessing two phenol rings linked to each other by an ethylene bridge. The chemical structure of resveratrol (*trans*-3,5,4′-trihydroxystilbene) is identified in two isomeric forms, *cis-* and *trans*-resveratrol (Figure 1). *trans* form is dominant in terms of its prevalence and different biological activities are attributed, namely in inducing cellular responses such as cell cycle arrest, differentiation, apoptosis, and to enhance cancer cells anti-proliferation [13,14,15].

Formal chemical name (IUPAC name) of resveratrol is *E*-5-(4-hydroxystyryl)benzene-1,3-diol. Various aspects on resveratrol chemistry are currently being studied. It exists as two geometric isomers: *cis*-(*Z*) and *trans*-(*E*). *trans* form can undergo to *cis* form isomerization when exposed to UV irradiation. *trans*-resveratrol powder was found to be stable under “accelerated stability” conditions of 75% humidity and 40 °C in the presence of air. The low resveratrol bioavailability was encumbered its therapeutic application. Therefore, modification of resveratrol structure has received special attention from researchers and many resveratrol derivatives have been synthesized such as methoxylated, hydroxylated and halogenated derivatives, all of them exhibiting favorable therapeutic potential [3,16,17]. Resveratrol is present in dietary products as glycosylated forms, known as piceid. Though, plants and pathogens, and even human digestive tract possess enzymes able to triggers polyphenols oxidation (and subsequent inactivation), the glycosylation prevents enzymatic oxidation of resveratrol, thereby preserving its biological effects and increasing its overall stability and bioavailability [18]. Furthermore, since intestinal cells can absorb only resveratrol aglycone form, absorption process requires glycosidases. Therefore, the relative aglycone and glycosylated resveratrol amounts in foods and beverages may modulate its absorption rate [19].

Three glycosylated resveratrol analogues, piceid, piceatannol glucoside, and resveratroloside isolated from the invasive plant species *Polygonum cuspidatum* [19] were identified as the major antibacterial compounds [20]. Glycosylated resveratrol analogues have comparable biological effects after transepitelial passage, as they can be hydrolyzed into deglycosylated forms, resveratrol in the intestine [21]. However, in vitro studies have shown that the glycosylated analogues even show more powerful bioactivities. For example, resveratrol and piceid have similar antioxidant capacity, but piceid appears to be more efficacious than resveratrol due to its reaction with its radical form [22,23]. Indeed, resveratrol-glycoside was more effective than resveratrol against hepatitis B virus [24,25]. Piceatannol, with one more hydroxyl group, was already reported as having stronger anti-inflammatory, immunomodulatory, anti-proliferative, anti-leishmanial, anti-leukemic, and protein-tyrosine kinase inhibitory effects [19].

Pterostilbene, a natural methoxylated resveratrol analogue, was first isolated from *Pterocarpus santalinus* (red sandalwood), a plant used in traditional medicine for diabetes treatment [26]. This *Pterocarpus marsupium* active constituent is mainly found in blueberries, grapes, and several plant woods. [26,27]. Pterostilbene has a similar structure to resveratrol except that in A ring 3 and 5 position was replaced by a methoxyl group [26]. This compound pro-lipophilicity, greater than that of resveratrol, increases its bioavailability [28,29,30] resulting in stronger bioactivities, including anticancer, anti-lipidemic, antidiabetic, and cardioprotective effects than those of resveratrol [26,31,32].

In the same line, resveratrol nanoformulation have been conceived as a promising approach for biological function retaining, where polycaprolactone form the hydrophobic core, whereas polyethylene glycol form the hydrophilic shell of the encapsulated resveratrol micelles [33,34]. Solid lipid nanoparticles and nanostructured lipid carriers are two unique resveratrol nanodelivery systems that were developed to enhance resveratrol’ oral bioavailability for nutraceutical purposes [35]. Indeed, resveratrol nanoparticles led to an improvement in its solubility and enhances its antioxidant potential than free form [35,36]. For example, resveratrol nanoformulation exhibited an in vivo absorption raise, length of action extension and bioavailability improvement by 3.516 times more, when compared with raw form [37]. In addition, the hydrophobic nature of resveratrol considerably contributes to its limited bioavailability, which results from its poor water solubility. Thus, resveratrol encapsulated in methylated-*β*-cyclodextrins (in a ratio 1:1) improved its water solubility (about 400-fold), and consequently its bioavailability, maintaining its antioxidant and antibacterial effects (against *Campylobacter*) [38], at same time that encourages its further application in food industry, aiming at foodborne pathogens control, as well as for nutraceuticals purposes.

## 3. Biological Activities of Resveratrol

Resveratrol possesses a wide range of biological properties, among them antioxidant, cardioprotective, neuroprotective, anti-inflammatory and anticancer activities [19,38].

### 3.1. Free Radical Scavenging and Antioxidant Effects

Resveratrol possess many biological properties, but the best described resveratrol property is their capacity to act as a potent antioxidant [39]. Resveratrol antioxidant activity depends upon the arrangement of functional groups on nuclear structure. Therefore, configuration, substitution, and total hydroxyl groups number substantially influence several mechanisms of antioxidant activity, such as radical scavenging and metal ion chelation abilities. Previous studies showed that hydroxyl group in 4′ position is not the sole determinant for antioxidant activity, but also the 3- and 5-OH groups [40,41]. The study of antioxidant effect against hydroxyl (^•^OH) and hydroperoxyl (^•^OOH) radicals in aqueous simulated media using density functional quantum chemistry and computational kinetics methods revealed that *trans*-resveratrol may act as an efficient ^•^OOH, and also presumably ^•^OOR, radical scavenger [42]. Resveratrol can also be used in minimizing or preventing lipid oxidation in pharmaceutical products, delaying toxic oxidation products formation, and maintaining both nutritional quality and prolonging pharmaceuticals shelf-life [43,44,45]. In addition, resveratrol’s antioxidant properties have been successfully employed to protect cells against hydrogen peroxide-induced oxidative stress, where the pre-treatment with resveratrol promoted cell survival and protection against UV-irradiation-induced cell death. Resveratrol cellular defense could be achieved, at least in part, by its ability to act as a direct antioxidant and an indirect cellular antioxidant system inducer through modulation of several cellular antioxidant pathways, thereby balancing cellular redox status [10,46,47].

As already highlighted, resveratrol is a powerful antioxidant that beneficial effect is hampered by its low bioavailability. Many attempts have been made to generate resveratrol derivatives by esterification process to improve their lipophilicity and application in lipid-based foods and biological environments. About 12 different esterified acyl chlorides have been synthesized including butyryl chloride, caproyl chloride, capryloyl chloride, capryl chloride, docosahexaenoyl chloride, eicosapentaenoyl chloride, lauroyl chloride, myristoyl chloride, oleoyl chloride, palmitoyl chloride, propionyl chloride, and stearoyl chloride. These derivatives were able to effectively inhibit copper ion-induced low-density lipoprotein (LDL) oxidation and inhibited hydroxyl radical-induced DNA scission [33]. These results clearly demonstrated that resveratrol derivatives might serve as potential antioxidants in foods and biological systems.

### 3.2. Anticancer Effects

Numerous studies have demonstrated that resveratrol possesses antitumor action and is a likely candidate for treatment and prevention several types of cancer [31,48]. The anticancer properties of resveratrol have been confirmed by many in vitro and in vivo studies, which show that resveratrol is able to inhibit all carcinogenesis stages (e.g., initiation, promotion and progression) [49,50,51]. Many studies also provided evidence that resveratrol not only acts a chemopreventive agent, but also display chemotherapeutic properties linked to its anti-inflammatory, antioxidant, pro-apoptosis and anti-proliferative actions [50,52]. Indeed, Resveratrol is believed to target intracellular signaling pathway components such as regulators of cell survival and apoptosis, pro-inflammatory mediators, and tumor angiogenic and metastatic switches by modulating a distinct set of transcription factors, upstream kinases, and their regulators [53]. For instance, resveratrol have demonstrated apoptotic and anti-proliferative effects on human cervical carcinoma by inducing cell shrinkage in HeLa cells and apoptosis through the activation of caspase-3 and -9, upregulation of the expression of the pro-apoptotic B-cell lymphoma (Bcl)-2-associated X protein and downregulation of the expression of the anti-apoptotic proteins Bcl-2 and Bcl-extra-large in HeLa cells, and increased expression of the p53, a protein that is essential for cell survival and cell cycle progression [54]. Cheng et al. demonstrate that resveratrol exert its anticancer action in in pancreatic cancer cells by suppressing the expression of NAF-1 through activation of Nrf2 signaling and inducing cellular reactive oxygen species accumulation that lead to apoptosis activation and prevent proliferation of pancreatic cancer cells [55]. Resveratrol is also an Histone deacetylase inhibitors that display its antiproliferative action by activating cell cycle arrest, inducing apoptosis and autophagy, angiogenesis inhibition, increasing reactive oxygen species generation causing oxidative stress, and mitotic cell death in cancer cells [56]. The presence of 4′-OH together with the stereoisomer in *trans*-conformation (4′-hydroxystyryl moiety) is absolutely required for cell proliferation inhibition [40]. Enzymatic assays demonstrated that DNA synthesis inhibition was induced by a direct interaction of resveratrol with DNA polymerases [40]. Another in vitro work has shown that resveratrol enhances chemotherapy effectiveness through inactivating NF-κB protein (a transcription factor) formed by cancer cells and which controls certain genes expression. When this factor is present, cancer cells become chemotherapy-resistant, which then allows them to multiply. Resveratrol acts blocking this transcription factor, thereby enabling chemotherapeutics to act at their targeted sites [57,58,59]. Resveratrol also attenuates the acetylation, phosphorylation, and nuclear translocation of NF-κB [60] and inhibit iNOS expression in colon cancer cells (a key enzyme in colon tumorigenesis induced by pro-inflammatory and cytokines agents) and the IGF-1R/Akt/Wnt pathways, and activates p53 to hampered cell and tumor development [60]. These effects fall into two classes: (i) Well-documented anti-proliferative and pro-apoptotic effects on cancer cell lines; and (ii) slightly more hypothetical chemopreventive effects that corresponds to resveratrol effects on cancer initiation [57,58,59].

Besides, the phytoestrogen, resveratrolt has received great attention as an upcoming preventive and therapeutic agent against breast cancer [61]. Resveratrol has also shown promise as part of combination therapy, particularly in breast cancer [62]. This compound has been shown to reverse drug resistance in a wide variety of in vitro cell systems by sensitizing tumor cells to drug-mediated effects in combination with other chemotherapeutic agents [50]. Resveratrol demonstrates ability to enhance the sensitivity of pancreatic cancer cells to gemcitabine therapy [55]. Cisplatin, a cancer chemotherapy agent against ovarian, bladder, testicular, and many other cancers, high risk of nephrotoxicity is reduce by Resveratrol [63]. Globally, many in vitro and animal-based studies have demonstrated such preventive anticancer activity in colon, cervical, prostate, breast and lungs [50,64,65,66,67,68,69]. Resveratrol-loaded nanoparticles have also demonstrated antioxidant potential in cancer cells [37]. In addition, resveratrol beneficial effects are also present when adopted as a conventional treatment support to cancer, using chemotherapy and radiotherapy [70,71,72]. Based on previous experimental and clinical trials, and on molecular characteristics of resveratrol, it could be used as: (i) A neoadjuvant chemotherapy agent before surgery to decrease tumor volume, owing to its ability to inhibit cancer cell proliferation and to induce apoptosis; (ii) an adjuvant chemotherapy drug to inhibit early cancer invasion and metastasis after surgery; (iii) a radiotherapy or chemotherapy sensitization agent in combination with chemotherapy agents, like capsaicin, docetaxel, doxorubicin, gemcitabine and temozolomide, since resveratrol may improve their anticancer effects; (iv) in cancer prevention for people under high risk of cancer; (v) a radioprotective agent to reduce treatment adverse effects, including radiotherapy-induced xerostomia and mucositis.

### 3.3. Cardioprotective Effects

Resveratrol protective effect was shown to improve cardiovascular function in diabetic rats [73,74] by preserving the functional abilities of cardiac stem/progenitor cell compartments and mature cardiac cells, improved cardiac environment by reducing inflammatory state and decreased unfavorable ventricular remodeling of the diabetic heart, leading to a marked recovery of ventricular function [74]. Resveratrol showed beneficial effect in heart failure by improving left ventricle function, decreased cardiac hypertrophy, contractile dysfunction and remodeling, interstitial fibrosis, and the level of plasma BNP [75]. Some molecular mechanism of resveratrol action include inhibition of prohypertrophic signaling molecules, improvement of myocardial Ca^2+^ handling, phosphorylation of prosurvival (Akt-1, GSK-3*β*) and stress signaling (MKP-1) pathways and the reduction of oxidative stress and inflammation (iNOS, COX-2 activity, and ROS formation) [75]. Yan et al. suggest that resveratrol act by preventing the expressions of endothelial nitric oxide synthase, vascular endothelial growth factor, and suppressing phosphorylation of p38 in rats with diabetes-related myocardial infarction [73]. Besides, resveratrol administration in myocardial infarction-related diabetic rats significantly reduced blood glucose, body weight, plasma triglyceride levels, heart rate and aspartate transaminase (AST)/alanine transaminase (ALT) ratio, at same time that markedly increased total plasma insulin levels [73,76]. In addition, resveratrol significantly reduced inflammation factors and malondialdehyde levels, which is a marker of oxidative stress [77]. These results showed that resveratrol treatment can improve cardiovascular function by reducing myocardial ischemia-reperfusion injury, vasodilation and atherosclerosis [78]. Contrarily, at physiological concentrations, resveratrol induces vasodilation, and consequently decreases hypertension and cardiovascular diseases risk [79]. On the other hand, these results have also confirmed the uses of *Polygonum cuspidatum* as a resveratrol source to treat and to prevent hyperlipidemia and arteriosclerosis in traditional chinese medicine [80,81,82]. Overall, the cardiovascular protective effect of resveratrol have been linked to multiple molecular targets and might be useful to the development of novel therapy for atherosclerosis, metabolic syndrome, ischemia/reperfusion, and heart failure [83].

### 3.4. Neuroprotective Effects

Resveratrol has several neuroprotective roles in various neurodegenerative impairments, such as Alzheimer′s, Huntington′s and Parkinson′s diseases, amyotrophic lateral sclerosis and alcohol-induced neurodegenerative disorders [84,85]. It has been shown that resveratrol protective effects are not limited to the anti-inflammatory and antioxidant activity but also improved mitochondrial functions and biogenesis through SIRT1(sirtuin 1)/AMPK/PGC1α pathway and vitagenes, which prevent the deleterious effects triggered by oxidative stress [85,86,87]. Resveratrol decreases cholinergic neurotransmission, brain-derived neurotrophic factor expression, and oxidative stress, promotes β-amyloid peptides clearance and anti-amyloidogenic cleavage of APP, and reduces neuronal apoptosis [88]. A meta-analysis showed that resveratrol significantly decreased Profile of Mood States (POMS) including vigor and fatigue but had no significant effect on memory and cognitive performance [89]. Among the isolated resveratrol oligomers, vitisin A and heyneanol A have been reported for better dose-dependent inhibitory potential compared with standard inhibitor (galantamine) on both acetylcholinesterase (AChE) and butyrylcholinesterase (BChE) activity [17,37]. Resveratrol is also able to improve rat motor abilities and to deactivate neuroinflammatory response following intracerebral hemorrhage. It may be used as a novel therapeutic agent to treat intracerebral hemorrhage [90,91].

### 3.5. Anti-Inflammatory Activity

Stilbenoids including resveratrol are non-nitrogenous polyphenols with acidic and amphiphilic characters with anti-inflammatory activity. Many of their targets are occurring on cyclooxygenase (COX), 5-lipoxygenase (5-LOX) and protein kinase B [92], which is associated with its ability to inhibit COX-1 and COX-2 activity along with transcription factors activity inhibition, directly involved in COX activity regulation [93]. Studies reported the ability of resveratrol to reduce the secretion and expression of inflammatory factors [94]. The anti-inflammatory activity of resveratrol prevents acute pharyngitis-induced inflammation by inhibiting NF-κB, tumor necrosis factor-α and interleukin-6 serum levels, macrophage inflammatory protein-2 and cyclooxygenase-2 activity levels, reactive oxygen species production and caspase-3/9 in rabbit models [94]. Resveratrol inhibit the ear oedema of mice, WBC and pleurisy exudates, decrease the production of NO, and elevate the activity of SOD in serum in acetic acid-induced pleurisy test, reduce the content of MDA and elevate the T-SOD activity in serum; RSV could inhibit the expressions of TP, PGE2, NO, and MDA in carrageenan-induced synovitis test supporting its analgesic and anti-inflammatory activities [95]. Resveratrol inhibit the activation of microglia that lead to the release of various pro-inflammatory factors, the production of reactive oxygen species, and the activation of signal pathways leading to neuroinflammation [96] in in vitro resveratrol modulates the inflammatory response at moderate to high concentrations within intestinal cells by down-regulating NF-κB activation and preventing mitochondrial dysfunction. This result was confirmed in vivo where resveratrol inhibits TNF-α production and NF-κB activation, decreases neutrophil infiltration in the intestinal mucosa, and represses intestinal tumorigenesis by regulating anti-inflammatory miRNA [97,98]. Chen et al. demonstrated that resveratrol significantly suppressed the TLR-4/MyD88/NF-κB signaling pathway in lysophosphatidylcholine-induced damage and inflammation that might be useful for treatment of arteriosclerosis [99]. Taken together, these studies suggest that resveratrol can prevent inflammation and oxidative stress, reduce the risk of carcinogenesis and developed as anti-inflammatory agent to improve the quality of life of patients.

### 3.6. Antimicrobial Activity

Resveratrol, in addition to the above described biological activities, has been studied for its ability to inhibit the growth of some pathogenic microorganisms, such as Gram-positive and Gram-negative bacteria and fungi [100]. Indeed, resveratrol has been shown to efficiently inhibit *Candida albicans* growth [101]. Dimethoxy resveratrol derivatives exhibited antifungal activity against *C. albicans* with minimum inhibitory concentration (MIC) values of 29–37 μg/mL, including against 11 other *Candida* species [102]. However, the putative candidacidal activity of resveratrol is a matter of controversy. In fact, a study indicates that resveratrol is not effective against both *C. albicans* and non-*C. albicans* species [101]. In another study, resveratrol antifungal activity against *C. albicans* could be reached at 400 μg/mL, thereby minimizing the antifungal role of resveratrol against *C. albicans*-caused infections [103].

*Campylobacter jejuni* and *Campylobacter coli* are the major causes of bacterial gastroenteritis, while *Arcobacter* species are also known to be human and animal pathogens. Resveratrol-hydroxypropyl-γ-cyclodextrin inclusion complexes improved resveratrol solubility and showed anti-*Campylobacter* and anti-*Arcobacter* effects. Furthermore, it inhibited biofilm formation and promoted biofilm dispersion even at sub-MIC concentrations and therefore could be developed as a new anti-biofilm agent to enhance foods shelf-life and safety [104].

Resveratrol showed antibacterial activity against Gram-positive bacteria and time-kill assays showed that its effects were due to its bacteriostatic action [105]. However, the mechanism underlying its antibacterial activity is not clearly understood [106]. Resveratrol was also able to affect cells with changes in cell morphology and DNA contents [105]. Hwang and Lim [106] demonstrated that resveratrol led to DNA fragmentation in *Escherichia coli*, inducing an SOS response; nevertheless, resveratrol also induced cell elongation without an SOS response and thereby inhibits bacterial cell growth by suppressing FtsZ (crucial for Z-ring formation) expression and Z-ring formation in *E. coli*.

From another point of view, reactive oxygen species (ROS), superoxide, peroxide, and hydroxyl radicals are thought to contribute to the rapid bactericidal activity of diverse antimicrobial agents. *E. coli* and *Staphylococcus aureus* culture supplemented with resveratrol and treated with antimicrobials reduced ROS concentrations to sublethal levels, that are mutagenic, while the absence of resveratrol allows ROS to high enough to kill mutagenized cells. Antimicrobial lethality suppression and mutant recovery promotion abilities evidenced by resveratrol suggests that this antioxidant may contribute to the emergence of several antimicrobials-resistant species, especially if new derivatives and/or resveratrol formulations markedly increase its bioavailability [107].

Pseudorabies virus is one of the devastating pathogen of swine for which there is no treatment and that often result in economic losses. Resveratrol showed antiviral activity by inhibiting the Pseudorabies virus replication and effectively increase the growth performance and reduce the mortality of Pseudorabies virus-infected piglets [108].

Pterostilbene is a methoxylated derivative of resveratrol that showed antibacterial activity against drug-resistant *Staphylococcus aureus* (MRSA) with minimum inhibitory concentration (MIC) superior of pterostilbene compared to resveratrol (8~16-fold). Pterostilbene anti-MRSA potency was related to bacterial membrane leakage, chaperone protein downregulation, and ribosomal protein upregulation and can be topically applied for treatment of skin MRSA infection bearing it less toxicity to mammalian cells [32]. Resveratrol is a potentially useful agent on *Staphylococcus aureus* pneumonia and *S. aureus*-induced infectious diseases treatment [109]. Also, resveratrol could alleviate rotavirus infection-induced diarrhea [109].

### 3.7. Other Biological Activities

Besides the cardioprotective, antioxidant, anticancer, neuroprotective, anti-inflammatory, anti-dyslipidemia, and antidiabetic effects of resveratrol, it also exhibits antiproliferative and androgen-lowering effects on theca-interstitial cells of ovary. Moreover, it exerts a cytostatic but not cytotoxic effect in granulosa cells, while inhibiting aromatization and vascular endothelial growth factor (VEGF) expression. These actions may be of clinical relevance in conditions associated with theca-interstitial cell hyperplasia, androgen excess, and abnormal angiogenesis, such as polycystic ovary syndrome. In addition, resveratrol may increase ovarian follicular reserve and prolong ovarian life span, serving as a potential anti-aging agent [110].

Resveratrol is also able to decrease histopathological and biochemical damages and to exert protective effects on ischemia-reperfusion injury induced ovarian damages. Resveratrol has become to continue a hotspot in many fields, including respiratory system diseases. Indeed, research has demonstrated that resveratrol is helpful in relieving pulmonary function in general population and plays a protective role in respiratory system diseases. The main protective effects of resveratrol in respiratory system diseases, including its anti-inflammatory, antiapoptotic, antioxidant, antifibrotic, antihypertensive, and anticancer activities were also examined. In resveratrol-treated patients, serum levels of certain biochemical markers (i.e., C-reactive protein, erythrocyte sedimentation rate, undercarboxylated osteocalcin, matrix metalloproteinase-3, tumor necrosis factor *alpha*, and interleukin-6) were also significantly decreased [111]. Therefore, the use of resveratrol as an adjuvant to conventional antirheumatic agents seems to be an optimum approach. Resveratrol can also be used as a protective and/or therapeutic agent, particularly in male infertility cases caused by testicular toxicity. On the other hand, resveratrol could be useful to protect health against several pathologies and ageing problems [84]. However, the comparative evaluation of animal and human studies shows that resveratrol cannot protect against metabolic diseases and their relevant complications. Nonetheless, it is important to point out that the clinical findings are influenced by many factors, such as sample size and study objectives. Till now, small sample size and high dosage levels were used to conduct most of clinical trials to assess resveratrol significance in chronic diseases [84]. Consequently, it is not easy to determine the exact safety range and therapeutic effectiveness of specific resveratrol doses on specific populations. In this sense, before prescribing resveratrol, patients should be properly advised for effective treatment with minimum side effects. Further evaluations are needed before declaring resveratrol as a beneficial compound for human health.

## 4. Negative Effects of Resveratrol

Resveratrol is widely known for its renowned beneficial biological effects, namely involving its chemopreventive and antioxidant properties. However, some studies have documented that it may behave as a pro-oxidizing agent [112]; thus, paradoxically, it may also have implication in pathology of several diseases.

Resveratrol antioxidant potential has been attributed to its ROS-scavenging capacity [112,113] and to an up regulation capacity on cells antioxidant defense [114]. Studies have reported that resveratrol could act as a signaling molecule within tissues and cells in modulating genes and proteins expression through redox-sensitive intracellular pathways activation. Thus, cell tolerance against oxidative environment could be attributed to gene expression changes and to a raise in antioxidant defense systems action and synthesis, which eventually results in cell survival and adaptation [115,116,117]. Moreover, depending on enzymatic reactions conditions, resveratrol can be (auto-)oxidized to generate semiquinones and relatively stable 4′-phenoxyl radical, finally leading to ROS production [118,119]. Such polyphenols’ oxidative reactions are influenced by pH and presence of hydroxyl anions or organic bases [120,121].

**Figure 2:**
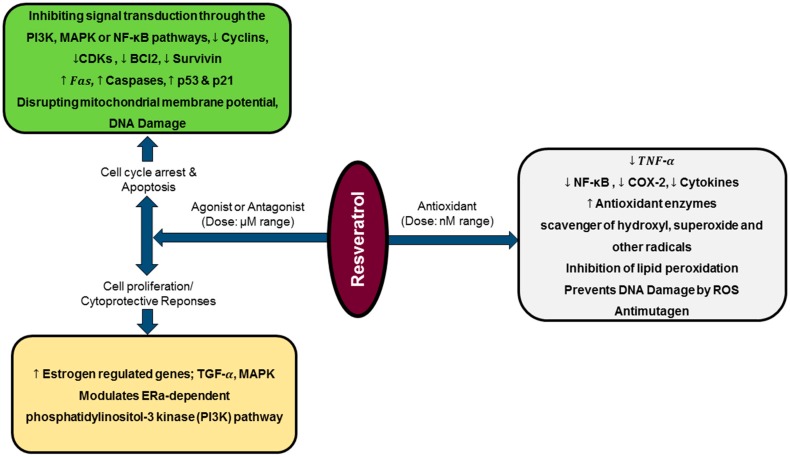
Diagrammatic representation of resveratrol biphasic activity and gene expression modulation. At nanomolar [124] doses, resveratrol acts as a potent antioxidant, while at micromolar (μM) range, it interacts as agonist or antagonist exhibiting cell proliferation/cytoprotective responses or cytostatic/apoptotic effects, respectively.

A study carried out by Martins et al. revealed that resveratrol can modulate different pathways at a time, which can result in distinct and even opposite biological effects, depending on its concentration or treatment time defined. The authors documented that, although a dose-dependent resveratrol pro-oxidative effect leads to cells oxidative stress over lesser time exposure, at same dose but with an increase in exposure time, less expressive cytotoxicity was found. This suggest that surviving cells seemed to be more resistant to resveratrol-induced damages, being its effects attenuated over treatment time [114]. Additionally, low resveratrol doses (0.1–1.0 μg/mL) has been documented to enhance cell proliferation, whereas higher doses (10.0–100.0 μg/mL) induces apoptosis (Figure 2) and decreases mitotic activity on human tumors and endothelial cells [122]. Recently, dual resveratrol pattern effects on HT-29 colon cancer cells death and proliferation were observed, where at low concentrations (1 and 10 μmol/L), resveratrol increased cells number, while at higher doses (50 or 100 μmol/L) resveratrol reduced cells number and increased apoptotic or necrotic cells percentage [123].

In a very interesting study, dose-time dependency of acute resveratrol administration on lipoperoxidation levels (in heart, liver and kidney of male rats synchronized with a 12-h dark-light cycle) was investigated. It was documented that resveratrol behaved as an antioxidant during dark span and as a pro-oxidant during light span, possibly reflecting the putative changing ratio between pro- and antioxidant activities in various organs during 24-h cycle or postprandial oxidative burst that occurred after a meal [124]. There is an interesting correlation among dietary polyphenols pro-oxidant and cytotoxic activities, such as to resveratrol. In fact, since every antioxidant is a redox agent it might become a pro-oxidant, accelerating lipid peroxidation and/or inducing DNA damages under special conditions. In this way, it has been proposed that such pro-oxidant action could be an important mechanism of action to resveratrol anti-cancer and apoptotic-inducing properties [112]. It has already been reported that resveratrol can lead to DNA damages, as well as to a reversible or irreversible cell cycle interruption mediated by its pro-oxidant effect [117]. Recently, Plauth et al. [125] proposed that cellular response to resveratrol treatment is based on oxidative triggering action, that can lead to cell fitness hormetic induction towards a more reductive state, so as to physiological resilience raising in fight oxidative stress. Also, it has been reported earlier that a critical balance between intracellular hydrogen peroxide (H_2_O_2_) and O_2_^–^ decides cells fate to apoptotic stimuli. Thus, a shift towards H_2_O_2_ favors apoptosis, whereas inclination towards O_2_^-^ obstructs apoptosis. Indeed, H_2_O_2_ promotes apoptosis by reducing intracellular O_2_^-^ concentration and triggering cytosolic pH drop. Ahmad et al. [126] reported that resveratrol inhibitory effect on H_2_O_2_-induced apoptosis is not due to its antioxidant activity, but rather, through a pro-oxidant effect evidenced by the prominent raise in intracellular O_2_^-^ production, which creates a non-conducive intracellular environment for apoptotic execution.

Regarding antioxidant/pro-oxidant hydroxystilbenes (resveratrol) activities, various studies were performed in the past aiming to define its structure–activity relationship, using cell-free systems [127,128]. Thus, Rüweler et al. [117] found that neither cytotoxic or cytostatic activities nor cytoprotective and antioxidant activities in cultured (C6 glioma) cells are indicative of a structure–activity relationship stressing the need to explore mechanisms at molecular level. Fukuhara and Miyata, firstly reported resveratrol pro-oxidant activity in a plasmid-based DNA cleavage assay, in the presence of transition metal ions, such as copper, the most redox-active metal ions present in nucleus, serum and tissues [129,130]. Resveratrol is closely linked with DNA bases, particularly guanine [131]. Copper ions from chromatin can be mobilized by metal-chelating agents, giving inter-nucleosomal DNA fragmentation rise, a property that is considered the hallmark of cells undergoing apoptosis. Recently, resveratrol mutagenicity in plasmid DNA was reported via point mutations (deletions/substitutions), resulting in many guanine bases deletion. In fact, since copper ions are known to be found in a nucleus bound to guanine bases in chromatin, the mobilization of such endogenous copper by resveratrol result in pro-oxidant DNA cleavage at the site. Moreover, copper concentration is reported to be raised in various malignancies; so, this study explains resveratrol anticancer activity [132].

Based on its structural similarity to diethylstilbestrol, a synthetic estrogen, resveratrol can also acts as a phytoestrogen, exhibiting variable estrogen receptor agonist degrees in different systems [133]. In some cell types, resveratrol acted as a super agonist, whereas in other ones, it produced an equal to or lesser activation than that of estradiol, and as an antagonist at higher concentrations. Such concentration-dependent agonist and antagonist behavior was employed to account for mechanisms underlying biphasic concentration response. At concentrations similar to those required for its other biological effects, resveratrol inhibited labelled estradiol binding to estrogen receptor and activated estrogen-responsive reporter genes transcription transfected into human breast cancer cells [133]. Besides, in absence of estrogen (E_2_), resveratrol exerts mixed estrogen agonist/antagonist activities in some mammary cancer cell lines, but in the presence of E_2_, resveratrol acts as an anti-estrogen [134]. In another report, it was demonstrated that resveratrol abolishes serum deprivation-induced elevated caspase 3 activity, suggesting its rescue effect via p38 MAPK signaling [135]. Resveratrol also regulates mitochondrial respiratory chain function, with mitochondrial complex I (CI) as a direct target of this molecule. It was also in vivo demonstrated that, in young and old mice brain mitochondria, resveratrol increased CI, while in aged animals with low antioxidant defenses led to oxidative stress. Therefore, not only dose, but also age at the time of treatment, can modulate intracellular and mitochondrial redox status, switching from resveratrol beneficial to deleterious effects, highlighting the importance of a balance between resveratrol pro- and antioxidant effects, that depends on its dose and age as well [136]. Yang et al. [137] reported dual resveratrol roles in pancreatic cancer cells: one as a tumor suppressor through Bax up-regulation, and the other one as a tumor activator through VEGF-B up-regulation; so, resveratrol anticancer effect is much stronger than cancer promotion effect.

All the above highlighted studies show the pivotal role of dose-dependency and aging in resveratrol-induced responses towards health benefits. Also, in another study, aiming to compare resveratrol effects on aging-induced and re-nutrition-induced insulin resistance and its consequences on arterial system, the authors found that resveratrol improved insulin sensitivity in old mice fed standard diet, while did not improve insulin resistance status in old mice receiving high-protein diets. In contrast, resveratrol exhibited deleterious effects by increasing inflammation state and superoxide production and decreasing aortic distensibility. This data demonstrates that resveratrol seemed to be beneficial to malnourished state of physiological aging, whereas when associated with high protein diets in old mice, may increase atherogenesis-associated risk factors by triggering vascular alterations that could represent an additional risk factor for cardiovascular system [138].

## 5. Side-Effects of Resveratrol

One of the most fascinating resveratrol aspects for its future development as a promising drug is that, it does not appear to have debilitating or toxic side effects. A wide range of resveratrol doses has been used in various in vivo and in vitro studies. However, it is imperative to find out the most appropriate dose and administration route. Also, it was documented that resveratrol induces cell death in tumor tissues with relatively no effect in normal adjacent tissues [52]. Resveratrol cell uptake disparity between normal and tumor cells may be attributed to differences in available cellular targets and gene expression in cancer cells, which makes resveratrol tumor-specific. Mukherjee et al. [139] have suggested that lower resveratrol doses could be associated with health benefits, while higher doses devastate tumor cells via pro-apoptotic effects.

Resveratrol does not appear to have side effects at short-term doses (1.0 g). Otherwise, at doses of 2.5 g or more per day, side effects may occurs, like nausea, vomiting, diarrhea and liver dysfunction in patients with non-alcoholic fatty liver disease [140]. Interestingly, no major side effects were stated in long-term clinical trials [141]. In fact, resveratrol has been found to be safe and well-tolerated at up to 5 g/day, either as a single dose or as fraction of multiple-day dosing schedule [142]. However, it is imperative to mention that these studies were done in healthy populations, and that may vary in sick patients. Our understanding of resveratrol dose-dependency and administration route is further complicated, since orally administrated resveratrol gets metabolized by gut microbiota [143], which makes it difficult to determine which effects are solely due to resveratrol or both resveratrol and its metabolites.

To investigate the assumption, whether resveratrol inhibit atherosclerotic development in hypercholesterolemic rabbits, Wilson et al. [144] supplemented rabbits with or without oral resveratrol (1mg/kg), and found that resveratrol treatment did not adversely affect rabbits health other than promoting atherosclerosis. Plasma LDL electrophoretic mobility was not different between groups. Atherosclerotic lesions staining in control and resveratrol-treated groups revealed that resveratrol-treated rabbits had significantly more aortic surface area covered by atherosclerotic lesions. Therefore, resveratrol promoted atherosclerotic development, rather than protect against it, by an independent mechanism of differences observed in gross animal health, liver function, plasma cholesterol concentrations, or LDL oxidative status [144]. Ferry-Dumazet et al. [145] aiming to analyze resveratrol nephrotoxicity effects, given orally 3000 mg/kg b.w. to rats for 28 days. It resulted in nephrotoxicity documented as elevated serum blood urea nitrogen and creatinine levels, increased kidney weights, gross renal pathology changes, and an increased incidence and severity of histopathological changes in kidneys. Kidneys microscopic evaluation identified lesions whose pathogenesis could be increased by resveratrol concentration (or its metabolite) as a function of renal osmotic concentration gradients, resulting in toxic levels in renal pelvis. This would result in necrosis, renal tubules obstruction and thus tubules dilation behind obstructed region. Indeed, inflammation and pelvic epithelium hyperplasia are expected responses to the presence of necrotic tissues. Therefore, administration of 1000 or 300 mg resveratrol/kg b.w./day did not result in nephrotoxic findings. The predominant clinical signs of toxicity at 3000 mg/kg b.w./day dose group were dehydration, piloerection, and red material in cage/urine, body weight gain reduction, hyperalbuminemia, anemia (due to renal injury, which reduced erythropoietin synthesis), white blood cell counts increase due to renal pelvic inflammation. Moreover, increased ALT, ALKP and total bilirubin levels suggest liver toxicity, but this was not histologically supported. Similarly, organs evidencing weight change did not evidenced histological changes [146].

Resveratrol has been reported to both reduce cell growth and induce apoptosis in normal cells, when administered at high doses, which confirm its biphasic effects over low to high concentrations spectrum [145]. Resveratrol rapidly activate mitogen-activated protein kinase (MAPK) in a MEK-1, Src, matrix metalloproteinase, and epidermal growth factor receptor in a dependent manner. It activates MAPK and endothelial nitric-oxide synthase (eNOS) at nanomolar concentrations (i.e., magnitude less than that required for ER genomic activity) and at concentrations possibly/transiently achieved in serum following oral red wine consumption [147]. Additionally, resveratrol consumption at modest doses result in a life span increase in 1-year old mice. However, when mice consumed larger resveratrol doses (1800 mg/kg), animals were shown to die within 3–4 months [148]. Studies on steady-state pharmacokinetics and tolerability of 2000 mg *trans*-resveratrol, administered twice daily with food, quercetin and alcohol (ethanol) showed that *trans*-resveratrol was well-tolerated by healthy subjects, although diarrhea was frequently observed [149].

## 6. Resveratrol Interactions: Drugs Perspective

### 6.1. Interaction with Cytochrome P450

The use of natural products is prevalent among patients who are taking conventional medicines, leading to a higher risk of natural product-drug interactions. Resveratrol may interact with several medications. It may lead to interactions with various cytochrome P450 (CYP), especially when taken at high doses [150]. Resveratrol has been reported to inhibit CYP3A4 activity, in vitro [151] and in healthy volunteers [152]. Therefore, high resveratrol intakes even in through form of supplements with additional medications could potentially reduce drugs metabolic clearance that undergo extensive first-pass CYP3A4 metabolism, hence increasing both bioavailability and toxicity risk of these drugs. Since this polyphenol has been reported to have significant interactions with phase I and II enzymes both in vitro and in vivo [153], they may be beneficial or harmful as well. Indeed, individuals taking drugs, such as tamoxifen, whose efficacy is highly specific and CYP enzymes-dependent, could be particularly affected. Therefore, caution should be taken when using supplemental resveratrol doses for health benefits, such as chemoprevention.

### 6.2. Interaction with Transporters

Aside from drug metabolizing enzymes, it is now greatly acknowledged that transport function modifications are involved in these resveratrol-drug interactions. Resveratrol has been reported to potently inhibit P-glycoprotein (P-gp), multidrug resistance-associated protein 2 (MRP2), and organic anion transporter 1/3 (OAT1/OAT3) [154]. Nonetheless, resveratrol interactions with transporters are still not fully elucidated. Furthermore, few clinical studies were conducted to determine transporter-mediated resveratrol-drug interaction. On the other hand, it is also speculated that higher resveratrol doses compete with other polyphenols for transporters, reducing both their uptake and potential synergistic effects. Moreover, absorption, distribution, renal excretion, and/or hepatic elimination of resveratrol active ingredients in humans is not well-explored than required for actual resveratrol-drug interactions prediction. Thus, resveratrol modulating effects on transporter-drug interactions warrants further investigation.

### 6.3. Interaction with Anticoagulant and Antiplatelet Drugs

Resveratrol has been reported to hinder human platelet aggregation in vitro [155,156]. Presumably, high resveratrol intakes in the form of supplements could enhance both bruising and bleeding risk when taken with anticoagulant drugs, antiplatelet drugs and even non-steroidal anti-inflammatory drugs (NSAIDs).

## 7. Conclusions and Future Perspectives

Resveratrol is a nutraceutical belonging to stilbenoid group, widely distributed in the plant kingdom and with several therapeutic effects. Structurally, stilbenoids possess two aromatic rings linked by an ethylene or ethene bridge with a variety of substituents. Even though, the presence of double bond suggests that stilbenoids exist in *cis*- as well as *trans*-form. *trans*-form is more stable and with high bioactive effects. Resveratrol molecules are synthesized from phenylalanine pathway through multiple enzymatic reactions. Traditionally, resveratrol has been used for stomachache, hepatitis, arthritis, urinary tract infections, fungal diseases or skin inflammation treatment, but the main biological potential of resveratrol belongs to cardioprotection.

Apart from its cardioprotective effects, resveratrol also exerts anticarcinogenic, antiviral, neuroprotective, anti-inflammatory and antioxidant properties. Resveratrol-like other derivatives are one of the most promising compounds on anti-inflammatory drug formulation. Nevertheless, its attractiveness, amendments to their structure/bioavailability/activity must be increased. Also, it has been shown that is able to mimic caloric restriction effects, exert anti-inflammatory and antioxidant effects, and even affect many diseases initiation and progression through several mechanisms. While there is a wealth of in vitro and in vivo evidence that resveratrol could be a promising therapeutic agent, clinical trials must confirm its potential.
